# Actinobacteria as Promising Biocontrol Agents for In Vitro and In Planta Degradation and Detoxification of Zearalenone

**DOI:** 10.3390/toxins16060253

**Published:** 2024-05-28

**Authors:** Larissa De Troyer, Noémie De Zutter, Sarah De Saeger, Frédéric Dumoulin, Siska Croubels, Siegrid De Baere, Leen De Gelder, Kris Audenaert

**Affiliations:** 1 Laboratory of Applied Mycology and Phenomics, Department of Plants and Crops, Faculty of Bioscience Engineering, Ghent University, 9000 Ghent, Belgium; 2 Centre of Excellence in Mycotoxicology and Public Health, Department of Bio-Analysis, Faculty of Pharmaceutical Sciences, Ghent University, 9000 Ghent, Belgium; 3 Laboratory of Pharmacology and Toxicology, Department of Pathobiology, Pharmacology and Zoological Medicine, Faculty of Veterinary Medicine, Ghent University, 9820 Merelbeke, Belgium; 4 Laboratory of Environmental Biotechnology, Department of Applied Biosciences, Faculty of Bioscience Engineering, Ghent University, 9000 Ghent, Belgium

**Keywords:** zearalenone, actinobacteria, biotransformation, degradation, detoxification, wheat, biocontrol

## Abstract

Zearalenone (ZEN) is a prevalent mycotoxin found in grains and grain-derived products, inducing adverse health effects in both animals and humans. The in-field application of microorganisms to degrade and detoxify ZEN is a promising strategy to enhance the safety of food and feed. In this study, we investigated the potential of three actinobacterial strains to degrade and detoxify ZEN in vitro and in planta on wheat ears. The residual ZEN concentration and toxicity in the samples were analysed with UHPLC-MS/MS and a bioluminescence BLYES assay, respectively. *Streptomyces rimosus* subsp. *rimosus* LMG19352 could completely degrade and detoxify 5 mg/L ZEN in LB broth within 24 h, along with significant reductions in ZEN concentration both in a minimal medium (MM) and on wheat ears. Additionally, it was the only strain that showed a significant colonisation of these ears. *Rhodococcus* sp. R25614 exhibited partial but significant degradation in LB broth and MM, whereas *Streptomyces* sp. LMG16995 degraded and detoxified ZEN in LB broth after 72 h by 39% and 33%, respectively. Although all three actinobacterial strains demonstrated the metabolic capability to degrade and detoxify ZEN in vitro, only *S. rimosus* subsp. *rimosus* LMG19352 showed promising potential to mitigate ZEN in planta. This distinction underscores the importance of incorporating in planta screening assays for assessing the potential of mycotoxin-biotransforming microorganisms as biocontrol agents.

## 1. Introduction

Zearalenone (ZEN) is a non-steroidal oestrogenic mycotoxin produced by several *Fusarium* spp. as a secondary metabolite and is chemically described as 6-[10-hydroxy-6-oxo-trans-1-undecenyl]-β-resorcyclic acid lactone. It exhibits an immuno-, hepato-, and genotoxic effect as well as a xeno-oestrogenic effect [1,2,3,4,5,6], with a similar chemical structure to the naturally occurring 17β-estradiol. Therefore, it can bind to oestrogen receptors and can cause hypoestrogenism after chronic exposure [7].

The main producer of ZEN is *Fusarium graminearum* (teleomorph *Gibberella zeae*). Additional species capable of producing this mycotoxin include *F. culmorum*, *F. crookwellense*, and *F. equiseti* [8,9,10]. It is commonly found in maize and small grains, such as barley and wheat, in association with another mycotoxin, namely, deoxynivalenol (DON) [11]. The transconfigured C1-C2 double bound of ZEN can be isomerised into a cis configuration upon the influence of UV or sunlight, resulting in another naturally occurring grain contaminant, namely cis-ZEN [12,13]. Plants have developed detoxification strategies to protect themselves from ZEN. These strategies comprise phase II conjugations with glucose or sulphate, resulting in zearalenone-14-glucoside (Z14G) and zearalenone-14-sulphate (Z14S), respectively [14,15]. Subsequently, these conjugated compounds are eliminated from the cytosol into the vacuole or irreversibly bound to the cell wall [14].

In animals and humans, ZEN is easily absorbed from the gastrointestinal tract into the bloodstream and metabolised in the liver, where it can undergo a phase I reduction of the C6 ketone to a hydroxyl group by 3α- and 3β-hydroxysteroid dehydrogenase, which leads to the formation of two stereoisomers, α- and β-zearalenol (ZEL), respectively [16]. The biotransformation ratio of ZEN to α- and β-ZEL differs between species. Given that α-ZEL exhibits higher oestrogenic activity while β-ZEL possesses lower oestrogenic activity compared to ZEN, these variations in metabolite transformation could account for the differences in susceptibility observed among different animal species [17,18]. Similar metabolisation and oestrogenicity profiles are observed for cis-ZEN [19,20]. Further reduction of the C11-C12 double bond results in α- or β-zearalanol (ZAL). ZEN and these reduced metabolites can also undergo phase II conjugations in the liver with glucuronic acid (glucuronidation) and, to a lesser extent, with sulphate (sulphonation). All these metabolites are excreted relatively rapidly in faeces, urine, and, to a small extent (less than 1%), in the milk of dairy cows [21,22].

Mycotoxins pose a significant threat to the safety of food and feed and, thus, to animals and humans through the consumption of contaminated grains. Therefore, EU directives were established that set maximum (guidance) levels of the most important mycotoxins, including ZEN, in wheat and derived products [23,24]. In order to not exceed these maximum values, significant degradation and detoxification strategies need to be implemented. Various physical, chemical, and microbiological methods to reduce the concentration of these mycotoxins have been developed, but the implementation of those physical and chemical methods is often costly and can negatively impact the level of essential nutrients in food and feed products [25,26,27]. Therefore, the microbiological transformation of ZEN is a promising alternative. In this paper, the term biotransformation is used to describe both the degradation and detoxification of ZEN.

Several studies have described the microbial transformation of ZEN, which have already been reviewed [28,29,30,31,32,33]. In these studies, members of *Bacillus* spp. and lactic acid bacteria are often reported to degrade and detoxify ZEN, along with species of *Streptomyces* and *Rhodococcus*. El-Sharkawy & Abul-Hajj (1988) investigated the degradation capacity of ZEN by *Streptomyces rimosus* and discovered a biotransformation of ZEN into 8′-hydroxy-zearalenone, which did not bind to a rat oestrogen receptor, indicating a loss of oestrogenicity [34]. Alkahtani et al. (2011) discovered four *Steptomyces* strains that can degrade ZEN in vitro, applied as a 1 mg/L standard, and produced by *F. verticillioides* [35]. Additionally, Krifaton et al. (2013) tested nine *Rhodococcus* spp. and one *Streptomyces* sp., which were all able to degrade ZEN, and most of them were also able to reduce oestrogenicity [36]. In a study by Cserháti et al. (2013), the potential of thirty-two *Rhodococcus* strains to transform five mycotoxins was investigated. Almost half of these strains, among which the nine *R.* strains used by Krifaton et al. (2013), could degrade ZEN with at least 14% efficacy [37]. Harkai et al. (2016) examined the capacity of ten *Streptomyces* spp. to transform ZEN, of which three showed significant degradation and detoxification [38]. In 2018, Risa et al. studied the degradation and detoxification potential of 42 type strains of the genus *Rhodococcus* and concluded that one strain, namely *R. percolatus* JCM 10087^T^, was able to degrade 95% of 1 mg/L ZEN and decreased oestrogenicity by 70% [39]. Lastly, Hu et al. (2023) identified the key degradation enzyme ZenR that is responsible for the degradation of ZEN by *R. erythropolis* HQ [40]. All these studies were performed in vitro in liquid growth medium, which bears little resemblance to the plant environment in which ZEN is actually to be remediated. Some studies investigated the biotransformation of ZEN on grain residues in vitro [41,42,43,44], and only a few studies have been performed on the biotransformation of ZEN in planta [45,46], but to our knowledge, no studies have been performed on the pre-harvest bacterial transformation of ZEN on wheat plants as a biocontrol strategy.

The aim of this study was to investigate the capacity of three actinobacterial strains, belonging to the genera *Streptomyces* and *Rhodococcus*, to biotransform ZEN. Firstly, their potential to transform ZEN was tested in vitro in LB medium as well as in a minimal medium (MM) with ZEN as the only carbon source. Secondly, we also investigated their biotransformation and survival potential in planta on detached wheat ears as an ecologically relevant matrix to where ZEN is found in a field setting.

## 2. Results

### 2.1. In Vitro Degradation and Detoxification of ZEN

Three actinobacterial strains (*S. rimosus* subsp. *rimosus* LMG19352, *R.* sp. R25614, and *S.* sp. LMG16995) were inoculated in a rich and minimal growth medium with 5 mg/L ZEN, and samples were taken every 12 h for three consecutive days (LB-medium) or every 4 days for eight consecutive days (MM) to monitor the degradation and detoxification of ZEN. Their potential to reduce the concentration of ZEN (degradation) was investigated by a targeted LC-MS/MS analysis. In order to assess the residual oestrogenicity of the samples (detoxification), a bioluminescence BLYES assay was performed. Additionally, an in vitro experiment with autoclaved cells was conducted to evaluate the impact of the adsorption of ZEN on the bacterial cell walls, which may cause a decrease in ZEN concentration and oestrogenicity that cannot be attributed to biotransformation.

#### 2.1.1. Degradation, Detoxification, and Adsorption of ZEN in LB Broth

**Figure 1:**
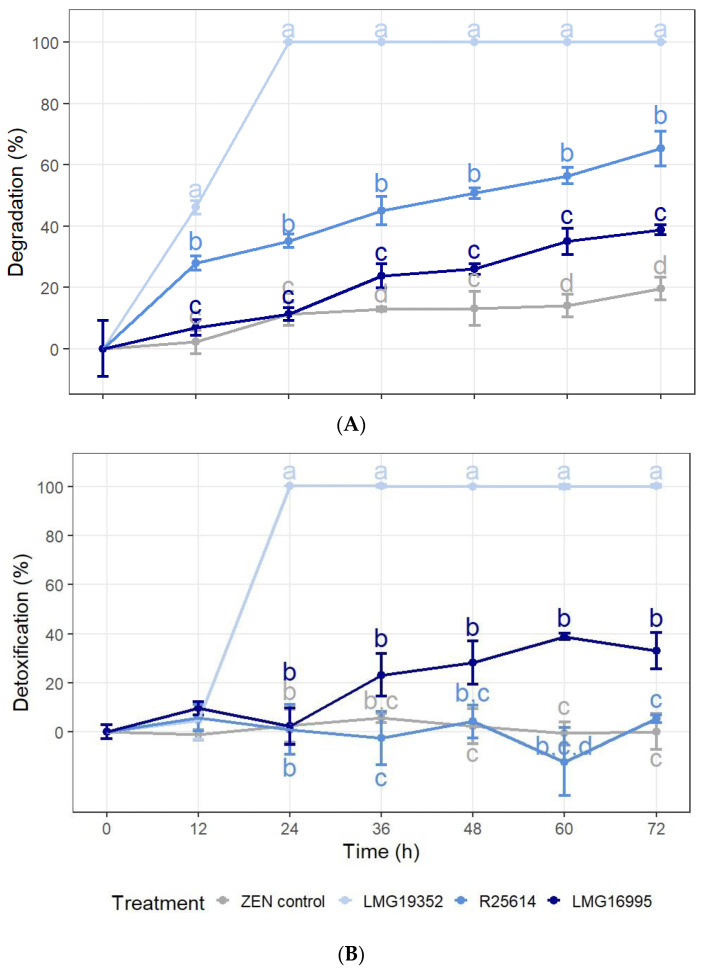
Percentage degradation (**A**) and detoxification (**B**) of 5 mg/L zearalenone (ZEN) by three actinobacterial strains in LB broth between 0 and 72 h after inoculation, analysed with LC-MS/MS and BLYES assays, respectively. Samples were incubated at 28 °C and 180 rpm. The values represent the means ± standard deviations (SD) of three replicates. Values not sharing common letters at a certain time point are significantly different (α = 0.05). When no significant differences were observed between treatments at a certain time point, significance letters were omitted.

The degradation and detoxification percentage of ZEN by three actinobacterial strains in LB was monitored throughout time (Figure 1) and repeated twice (Figure S1). After 60 h and 72 h of incubation in LB broth, all three strains showed a significant degradation of ZEN as compared to the uninoculated ZEN control (Figure 1A). A significant reduction of ZEN was already observed after 12 h for strains LMG19352 and R25614, which was not due to adsorption to the biomass as no loss in oestrogenicity was detected in the aliquot samples of autoclaved cells (Table S1). A complete degradation of 5 mg/L ZEN by strain LMG19352 occurred within 24 h, while strains R25614 and LMG16995 reached their maximum degradation percentages after 72 h of, respectively, 65% and 39%. In none of the samples, α-ZEL, β-ZEL, α-ZAL, or β-ZAL were detected above the detection limit. No ZEN was detected in the blank samples.

The results of the BLYES assay show that after 72 h, strains LMG19352 and LMG16995 were able to significantly detoxify ZEN (Figure 1B). Strain LMG19352 completely detoxified ZEN between 12 h and 24 h of incubation, while strain LMG16995 generated a 23–39% detoxification after 24 h. Throughout the experiment, strain R25614 did not show any significant differences compared to the ZEN control, indicating that this strain is not able to detoxify ZEN in LB broth. No cytotoxic effects were detected in the samples with *S. cerevisiae* BLYR.

#### 2.1.2. Degradation and Detoxification of ZEN in MM with ZEN as the Only Carbon Source

**Figure 2:**
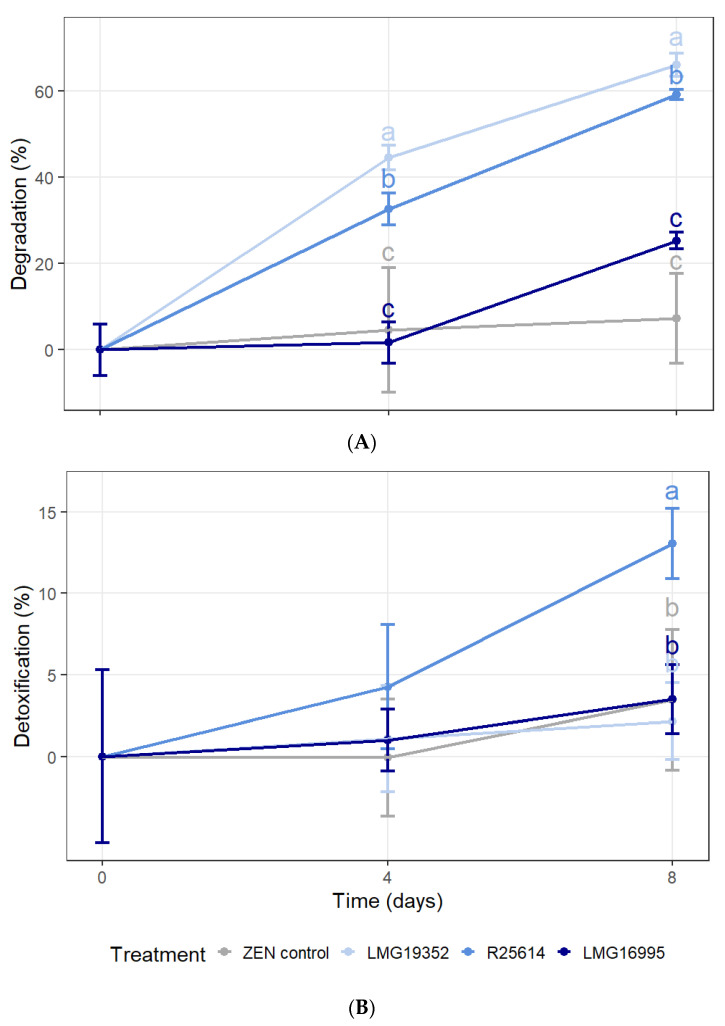
Percentage degradation (**A**) and detoxification (**B**) of 5 mg/L ZEN by three actinobacterial strains in MM with ZEN as the only carbon source at different time points, analysed with LC-MS/MS and BLYES assays, respectively. Samples were incubated at 28 °C and 180 rpm for 8 days. The values are the means ± SD of four replicates. Values not sharing common letters at a certain time point are significantly different (α = 0.05). When no significant differences were observed between treatments at a certain time point, significance letters were omitted.

The biotransformation of ZEN by three actinobacterial strains in MM was investigated over time (Figure 2) and repeated twice (Figure S2). The results from the LC-MS/MS analysis show that, after 4 and 8 days, strains LMG19352 and R25614 were able to significantly degrade 5 mg/L ZEN in MM (Figure 2A). These results indicate that both strains can use the mycotoxin as a carbon source for energy and growth. On the other hand, strain LMG16995 was not able to significantly reduce the ZEN concentration, although a slight degradation of 25% was measured after 8 days of incubation. Since it was observed that strain LMG16995 grew slower than the other two strains, it is plausible to assume that ZEN can also be used as a carbon source by this strain, but at a slower rate. In none of the samples, α-ZEL, β-ZEL, α-ZAL, or β-ZAL were detected above the detection limit, and no ZEN was detected in the blank samples.

The results from the BLYES assay show that only strain R25614 was able to significantly reduce the oestrogenicity of the samples by 13% after 8 days, while on day 4, none of the strains showed a reduced oestrogenicity compared to the ZEN control (Figure 2B). No cytotoxic effects were detected in the samples with *S. cerevisiae* BLYR.

### 2.2. Colony-Forming Units of Actinobacteria on Wheat Ears

To investigate whether the actinobacterial strains could survive on wheat ears for a longer period of time, they were inoculated on wheat ears. After 25 days post-inoculation (dpi), the samples were plated out on agar plates to count the number of colony-forming units (CFUs) (Table 1). For all three strains, CFUs were observed, indicating they can survive for several weeks on this ecologically relevant wheat matrix, although the number of CFUs of strains R25614 and LMG16995 was not significantly different from 0.

### 2.3. Degradation of ZEN on Wheat Ears

In order to assess the potential of these actinobacteria to successfully degrade ZEN under ecologically more relevant conditions compared to the in vitro experiments, detached wheat ears were inoculated with a 5 mg/L ZEN standard and a bacterial suspension of one of the three previously investigated actinobacteria. After 5 days of incubation, samples were analysed using LC-MS/MS to evaluate the degradation of ZEN on wheat ears. Control samples were taken on day 0 and day 5.

The absolute ZEN concentration in the wheat ears of the control samples in ng ZEN per spikelet on day 0 and day 5 was, respectively, 5 and 27 times lower than the expected concentration of 50 ng ZEN/spikelet (Table 2). Our hypothesis states that ZEN gets lost during the sample preparation, more specifically during the drying and crushing of the wheat samples, which would explain the loss at the beginning of the experiment, right after ZEN is spiked on the ears. The additional reduction in ZEN concentration on day 5 can possibly be explained by the transformation of ZEN into Z14G and Z14S by the plant [14,15], but these metabolites were not included in the LC-MS/MS method to verify. Furthermore, hidden ZEN might have been formed after 5 days by non-covalent interactions with matrix constituents, reducing extraction efficiency and thus resulting in a decreased detection of ZEN. This phenomenon has already been observed in maize, where ZEN can bind to the main protein, zein, due to the formation of hydrogen bonds and hydrophobic and electrostatic interactions [47]. Although similar observations for wheat are not yet available in the literature, this might have played a role in our experiment. Since heat and alkaline treatments are shown to result in the conversion of zein-bound ZEN into free ZEN [48], performing an additional alkaline or heating step during mycotoxin extraction might increase the content of detected ZEN in wheat samples as well. Alternatively, the additional loss on day 5 could be explained by a shift in the microbiome on the ears. Since the wheat ears used during the experiment were not sterile and ZEN is produced by *Fusarium* spp. that mainly colonise ears, it is not unlikely that other ZEN-degrading microorganisms occur in this niche and that ZEN is naturally degraded by those organisms.

**Figure 3:**
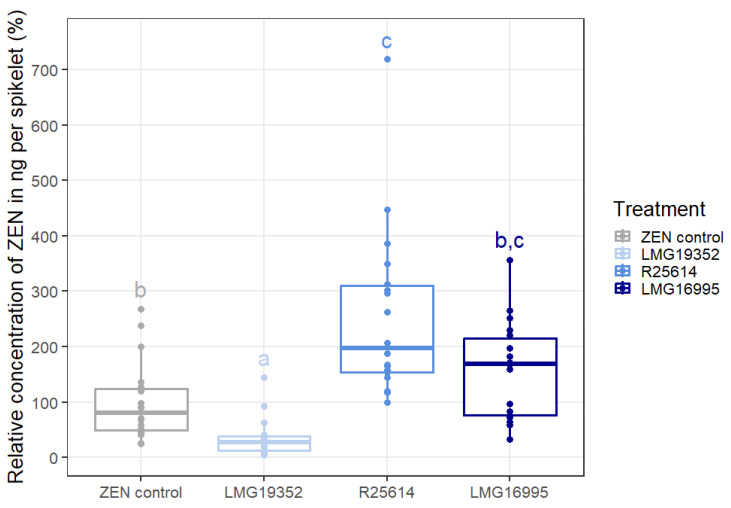
Relative residual concentration of ZEN in ng per spikelet (%) compared to the ZEN control (1.84 ng/spikelet = 100%) by three actinobacterial strains on detached wheat ears, analysed with LC-MS/MS. Boxplots indicate the median (horizontal lines), 25th and 75th percentile ranges (boxes), and up to 1.5× interquartile ranges (whiskers) (*n* = 18 biological replicates, analysed in two separate batches of 9 biological replicates). Values not sharing common letters are significantly different (α = 0.05).

In order to assess the impact of the actinobacteria on the degradation of ZEN on wheat ears, the relative residual concentration of ZEN in ng per spikelet compared to the ZEN control on day 5 is shown (Figure 3). It can be concluded that, of all three bacterial strains, only strain LMG19352 was able to significantly reduce the concentration of ZEN on wheat ears by 49% as compared to the ZEN control. An important consideration in this assay is the use of acetonitrile as the solvent of the ZEN standard, which might have negatively impacted the microorganisms growth. Although this effect is suggested to be minimal due to the rapid evaporation of the solvent, it could result in an underestimation of the beneficial effect of the actinobacterial strains. The other two strains, R25614 and LMG16995, resulted in a higher ZEN concentration compared to the ZEN control. A possible explanation for these results follows again from the assumption that the wheat ears are naturally colonised by ZEN-degrading microorganisms that can efficiently degrade ZEN. The introduced actinobacterial strains will be able to compete with these naturally occurring ZEN-degrading microorganisms for space and nutrients, which can eventually lead to a lower degradation level than in the control treatment. Another possible explanation for these results, is that the shift in the microbiome by strains R25614 and LMG16995 stimulated the growth of mycotoxin-producing fungi, and therefore a higher ZEN concentration was measured. Since we did not detect α-ZEL, β-ZEL, α-ZAL, or β-ZAL during the in vitro experiments, we did not include these metabolites in the method of our in planta experiment. There was no ZEN detected in the blank controls.

A BLYES assay could not be performed on the wheat ear extracts. The concentration of ZEN in these samples (Table 2) is lower than the lowest observed effect concentration (LOEC) (=0.03 µg/mL or 10 nM) [36], and the sample size is not big enough to upconcentrate, plus the internal standard zearalenone (ZAN) in these extracts has a synergistic oestrogenic effect with ZEN [47], making it impossible to correct for this factor when we would try to upconcentrate the samples.

## 3. Discussion

The microbial degradation and detoxification of toxic fungal metabolites within plant material offers a promising and innovative avenue for mitigating the risks associated with human and animal exposure to mycotoxins. To evaluate the overall potential of three actinobacterial strains (*Streptomyces rimosus* subsp. *rimosus* LMG19352, *Rhodococcus* sp. R25614, and *Streptomyces* sp. LMG16995) to degrade and detoxify ZEN, a sequential in vitro and in planta approach was pursued.

The in vitro part is composed of two different media, a rich medium and a minimal medium (MM), with ZEN as the only carbon source, to provide a better insight into the mechanisms through which these actinobacteria degrade and detoxify ZEN. The biotransformation of ZEN in LB broth suggests that ZEN is transformed by co-metabolism. Co-metabolism is a metabolic phenomenon in which an organism metabolises a compound without using it as a primary carbon or energy source. Instead, this compound is accidentally transformed by an enzyme or cofactor as a result of the microbial metabolic activity of another substrate. Typically, this process does not confer any clear benefit to the involved microorganism [48]. Conversely, in MM, it is assumable that, during the degradation of ZEN, the mycotoxin serves as a primary carbon source for the growth and energy production of the actinobacterial cells, since it is the only carbon source available in this medium.

After the in vitro screenings, the strains were challenged to perform ZEN degradation in planta to assess their effectiveness under ecologically more relevant conditions. Given the substantial differences between field conditions and those used in in vitro studies, it is not unlikely that good degrading organisms in vitro would fail to deliver under field conditions. In spite of that, state-of-the-art research regarding the microbiological degradation of ZEN still often only focuses on the degradation in vitro [34,35,36,37,38,39,40]. We want to emphasise the pivotal role of in planta validation in order to present strains that have a higher potential to be successful in a field application. When successful, the need for post-harvest remediation additives such as mycotoxin binders and modifiers like ZENzyme^®^ (Biomin^®^) [49] will be diminished.

### 3.1. Streptomyces rimosus subsp. rimosus LMG19352

*Streptomyces rimosus* subsp. *rimosus* LMG19352 acted as the best performing strain for both degradation and detoxification of ZEN in LB broth, since it was able to completely degrade and detoxify 5 mg/L ZEN within 24 h. Similarly, in a study by Krifaton et al. (2013), the investigated *Streptomyces* strain exhibited the best performance of all tested strains, resulting in both 100% degradation and detoxification of ZEN after 3 days [36]. Additionally, Harkai et al. (2016) noted that two *Streptomyces rimosus* strains (K145 and K189) completely degraded and detoxified 1 mg/L ZEN in LB within 5 days, while other *Streptomyces* spp. in their study were less effective [38]. In our study, we observed a reduction in ZEN concentration of 46% already after 12 h of incubation, while a delayed significant detoxification of ZEN occurred after 24 h. These results indicate that this strain first transforms ZEN into another highly oestrogenic compound that acts as an intermediate product, which is later degraded into a non-oestrogenic compound. A somewhat comparable metabolisation was reported by Krifaton et al. (2013) for various *R. pyridinivorans* strains, namely AK37, K408, and K402. While they did not establish a comprehensive time-lapse degradation profile for these strains, focusing solely on detoxification kinetics, it is noteworthy that they observed a fluctuating pattern in oestrogenicity. Initially, there was a decrease in toxicity, followed by an increase, then another decrease [36], suggesting the production of a highly oestrogenic intermediate compound by these strains.

In contrast, when grown in MM, strain LMG19352 degraded 66% of 5 mg/L ZEN after 8 days but did not show any detoxification, suggesting the transformation of ZEN into a highly oestrogenic end-product, which was reported before by El-Sharkawy et al. (1988) for two other *Streptomyces* spp., namely *S. griseus* ATCC 13,273 and *S. rimosus* NRRL 2234 [34]. The results of both our in vitro experiments imply that ZEN can be transformed by strain LMG19352 to serve as a carbon source for growth and energy, but that the resulting by-product remains highly estrogenic. Possibly, this by-product can only be transformed into a non-oestrogenic compound in LB broth through co-metabolism, which could explain why we only observed a reduced oestrogenicity in LB broth.

Furthermore, strain LMG19352 reduced 49% of the ZEN concentration within 5 days on detached wheat ears as compared to the ZEN control. Although the degradation rate of strain LMG19352 on wheat ears was similar to that in MM, it was lower than in LB medium. A somewhat similar phenomenon was observed for another bacterial genus by Xu et al. (2021), where *Bacillus amyloliquefaciens* MQ01 degraded ZEN in vitro in naturally contaminated corn at a lower rate compared to a standard solution in MM and suggested this effect might be due to the more difficult accessibility of ZEN in this complex matrix [41]. Despite the fact that ZEN was added as a standard solution in our in planta experiment, this might have played a role. Additionally, we assume that the bacterial growth rate on wheat ears is lower than in LB broth, resulting in a lower degradation rate, although strain LMG19352 demonstrated continued growth on wheat ears even 25 days after inoculation. Nevertheless, this is the first report of successful bacterial ZEN degradation on wheat plants, and overall, it can be concluded that the implementation of *S. rimosus* subsp. *rimosus* LMG19352 is a promising biological method to mitigate ZEN contamination. Moreover, Tan et al. (2021) investigated the biocontrol capacity of strain LMG19352 against *F. graminearum* and *F. poae* and showed a direct antagonistic effect against both fungi and a reduction in DON production on wheat ears [50]. Their results, together with the results in this research paper, designate strain LMG19352 as a promising pre-harvest biocontrol agent against *F. graminearum* and its mycotoxins.

### 3.2. Rhodococcus sp. R25614

*Rhodococcus* sp. R25614 was capable of degrading over 65% of 5 mg/L ZEN in LB broth, which did not result in a reduction in oestrogenicity, suggesting the transformation of ZEN into another, unidentified highly oestrogenic compound that cannot be further converted. This observation aligns with findings from prior research by Cserháti et al. (2013) on *R. globerulus* N58 and *R. aetherivorans* AK44, which were both able to degrade approximately 30% of 2 mg/L ZEN in LB after 72 h but failed to detoxify it [37].

Contrastingly to the results in LB broth, strain R25614 was able to both degrade and detoxify 5 mg/L ZEN in MM by 59% and 13%, respectively. This indicates that ZEN can be used as a carbon source and is (partially) transformed into a less oestrogenic compound, which has been reported before in other genera by Krifaton et al. (2013). They reported that *Gordonia paraffinivorans* NZS6 degraded 98% of ZEN by HPLC analysis, yet detoxified only 40% of the oestrogenicity by BLYES, indicating that this strain converts ZEN into a less yet still oestrogenic compound as well.

From the results of both in vitro assays, it can be concluded that if ZEN is used in MM, it is partially detoxified, but if ZEN is used in LB broth, it does not lead to detoxification. This suggests that strain R25614 can utilise ZEN both as a carbon source and through co-metabolism, but potentially by using a different biotransformation pathway. Alternatively, it is possible that strain R25614 uses ZEN as a carbon source in LB broth and transforms it into another highly oestrogenic by-product, but prioritises using the alternative available carbon sources, resulting in no further degradation of this by-product and consequently no detoxification in the samples. In a study by Yu et al. (2011), somewhat comparable results were observed in another bacterial genus. They discovered that *Acetinobacter* sp. SM04 degraded and detoxified ZEN in MM with ZEN as the only carbon source but not in rich nutrient broth (NB), indicating that strain SM04 prefers metabolising alternative carbon sources when they are available rather than ZEN [51]. It needs to be noted that partial detoxification in LB but no detoxification in MM was observed for strain R25614 during the repeated experiment (Figures S1 and S2), indicating that this strain is not consistent in detoxifying ZEN and implying that the conclusions above are not conclusive.

On wheat ears, no degradation of ZEN was observed by strain R25614 after 5 days. Since the number of CFUs of this strain was not significantly different from zero after 25 days of inoculation, it is possible that strain R25614 does not survive well on wheat ears, even within a period of 5 days, and therefore cannot exhibit its degradation capacity in vitro. Although it is desirable for these degrading microorganisms to survive for a longer period of time (ideally from Zadoks Growth Scale (GS) 55 until GS 92), performing a survival test at 5 dpi could provide a more conclusive evaluation of their viability, which is responsible for their inability to biotransform ZEN on wheat ears. During this survival test, the impact of the solvent in which the ZEN standard was dissolved (acetonitrile (ACN)) on the survival of the actinobacterial cells should be taken into account as well.

### 3.3. Streptomyces sp. LMG16995

*Streptomyces* sp. LMG16995 could both degrade and detoxify ZEN in LB broth, albeit to a lesser extent than strain LMG19352. At 36 h, a difference with the ZEN control was measured in regard to both degradation and detoxification, although the difference concerning detoxification was not significant. Moreover, both degradation and detoxification increased by 23–39% over time, suggesting that the degradation profile of this strain has a similar pattern as the detoxification profile and thus indicates a direct conversion of ZEN into a less toxic compound. In a study by Harkai et al. (2016), partial degradation and detoxification of ZEN by *Streptomyces* were reported for three other strains, namely *S. violaceoruber* K136, *S. sanglieri* K139, and *S. cacaoi* subsp. *asoensis* K234. Although it needs to be noted that the supernatant of autoclaved cells from the latter one showed a significant reduction in oestrogenicity, implying the loss in ZEN concentration is partially due to adsorption instead of biotransformation, which was not observed in our study. Moreover, Harkai et al. (2016) only monitored the degradation and detoxification of ZEN at the end of their experiment; thus, no comparison of their degradation or detoxification kinetic profile over time is possible [38]. It needs to be noted that no significant detoxification was observed during the repeated experiment (Figure S1), indicating that the detoxification potential of strain LMG16995 in LB broth is not consistent and implying that the conclusions above are not conclusive.

In MM, strain LMG16995 showed no degradation; hence, no detoxification was observed in the BLYES assay, implying that ZEN cannot be used as a carbon source by this strain and undergoes transformation through co-metabolism in LB broth. These results can somewhat be supported by a study by Ahad et al. (2017), where no detoxification of DON was observed by a microbial consortium (DX100) in MM, although detoxification of DON occurred in rich media such as NB and LB [52].

On wheat ears, similar to strain R25614, no degradation of ZEN was determined by strain LMG16995 after 5 days, and no significant survival was measured after 25 days, which might explain these findings. Performing a survival test at 5 dpi and investigating the impact of ACN on the growth could also provide a more solid conclusion.

### 3.4. Future Perspectives

Although the outcome of our study is promising, further scientific research is needed before these actinobacterial strains can be implemented in the field.

To start, it would be interesting to establish a detoxification profile of ZEN during our in planta experiment to verify whether the degradation of ZEN by strain LMG19352 also results in reduced oestrogenicity. Increasing the sample size and working with extracts without oestrogenic internal standards such as ZAN can eliminate biases associated with the BLYES assay. According to Yu et al. (2011), oestrogenic effects after biotransformation could alternatively be detected with a more sensitive toxicity assay based on a methyl thiazole tetrazolium (MTT) cell proliferation assay in MCF-7 cells [51,53].

Secondly, identifying the degradation products could give us a better understanding of the mechanisms underlying ZEN degradation and detoxification. An HPLC-analysis could provide a first insight into which metabolites are formed during the degradation of ZEN based on retention time, whereas a full MS scan could provide similar information based on mass-to-charge ratio and retention time. Identification of these metabolites is only possible after conformation with analytical standards. Alternatively, the identification of these degradation products could be achieved by more high-end techniques such as analysing ^13^C labelled ZEN via Nuclear Magnetic Resonance (NMR) spectroscopy or via untargeted metabolic profiling that combines stable isotopic labelling (SIL) and LC-HRMS. These methodologies have been previously employed by other researchers to study the transformation of several mycotoxins, such as DON [54,55,56], fumonisin B1 [57], HT-2 toxin, and T-2 toxin [58,59].

Thirdly, further research is needed to explore the capability of our actinobacterial strains to degrade and detoxify naturally produced ZEN by *F. graminearum*, since the performance of the bacterial strains may vary in the presence of the fungal pathogen. Previous research within our group, performed by Tan et al. (2021), showed that the direct biocontrol effect of the most promising strain in our study, LMG19352, against the virulent *F. graminearum* was reduced in the presence of the weakly pathogenic *F. poae*. This result implies the biocontrol potential and hence the co-existence of strain LMG19352 together with other *F.* spp., although the effect on the degradation of ZEN in the presence of *F. graminearum* was not investigated in his study [49]. Other studies regarding the degradation of naturally produced ZEN have been conducted earlier by Alkahtani et al. (2011), El-Naggar et al. (2012), Abdallah et al. (2018), and Gimeno et al. (2020). The in vitro degradation of various mycotoxins, including ZEN, produced by *F. verticillioides, F. solani*, and *F. oxysporum* was investigated by Alkahtani et al. (2011) and El-Naggar et al. (2012). They both concluded that all tested *Streptomyces* strains were effective in reducing the concentration of ZEN [35,60], although this reduction might originate from a decrease in fungal biomass and thus less production of ZEN instead of biotransformation. This issue was addressed by Abdallah et al. (2018), who visualised the concentration of ZEN relative to the growth of *F. graminearum* in vitro but also studied the degradation of ZEN on maize plants by several fungal endophytes [45]. In a study by Gimeno et al. (2020), the concentration of ZEN in harvested wheat grains was analysed after treating a wheat field with *F. graminearum*-infected maize stalks inoculated with the fungal biocontrol agents *Clonostachys rosea* or *Trichoderma atrobrunneum*. They observed a significant reduction in ZEN concentration in all treatments compared to the control treatment in 2018, although this reduction is possibly not attributable to biotransformation since they detected a decrease in Fusarium Head Blight (FHB) severity and biomass of *F. graminearum* [46]. Performing an in planta experiment with a GFP-labelled *F. graminearum* makes it possible to distinguish between the reduction of ZEN due to biotransformation or a reduction in fungal biomass, which was conducted by other researchers in our lab [50,61].

Finally, future research comprising field trials should investigate the effect of photoisomerisation of trans-ZEN into cis-ZEN, since the degradation and detoxification potential of our strains may be altered in the presence of cis-ZEN. In a study by Dellafiora et al. (2015), it was described that the trans-ZEN detoxifying enzyme ZHD, derived from *Clonostachys rosea*, differently interacts with cis-ZEN compared to trans-ZEN [62].

## 4. Conclusions

In the present study, three actinobacterial strains were evaluated for their potential to degrade and detoxify 5 mg/L ZEN in vitro and in planta on wheat ears. *Streptomyces rimosus* subsp. *rimosus* LMG19352 exhibited the best performance of all three strains in both in vitro tests as well as in planta. It was effective in completely degrading and detoxifying ZEN in LB broth within 24 h and degrading over 60% in MM after 8 days. Additionally, it was able to degrade 49% of ZEN on wheat ears and could survive on these ears for at least 25 days, indicating it is a good ear coloniser, which is desirable for field implementations. *Rhodococcus* sp. R25614 could degrade ZEN in LB broth and in MM, although (almost) no detoxification was observed. As transformation products derived from the conversion of ZEN still might be highly estrogenic, it highlights the importance of detoxification assays such as the BLYES assay. *Streptomyces* sp. LMG16995 could degrade and detoxify ZEN in LB broth, but not in MM or on wheat ears. Overall, these findings underscore the potential of actinobacteria for effectively mitigating ZEN contamination, although further research in this area is needed for agricultural and food safety applications. Moreover, future studies evaluating the toxicity and even the structure of the biotransformation products will help to explain the mechanisms and evaluate the biosafety for field implementation of these bacteria.

## 5. Materials and Methods

### 5.1. Actinobacterial Strains

Three actinobacterial strains were used in this study, namely *Streptomyces rimosus* subsp. *rimosus* LMG19352, *Rhodococcus* sp. R25614, and *Streptomyces* sp. LMG16995 from the Belgian Coordinated Collections of Microorganisms (BCCM) (Ghent, Belgium). They were grown from their −80 °C glycerol stock on tryptic soy agar (TSA) plates for 3 days at 28 °C, after which a single colony was picked with a sterile inoculation loop and grown in 5 mL tryptic soy broth (TSB) for 3 days at 28 °C and 180 rpm. The bacteria were then centrifuged at 4000 rpm for 5 min and washed twice with Phosphate Buffered Saline (PBS). Eventually, the optical density at 600 nm (OD_600_) was measured with a spectrophotometer and set to a value of 0.6.

### 5.2. Chemicals and Mycotoxin Standards

A solid standard (5 mg) of ZEN, used during the in vitro biotransformation experiments, was purchased by Merck (Hoeilaart, Belgium) and dissolved in methanol at a concentration of 1000 µg/mL and stored at −20 °C. A certified ZEN standard (5 mL) of 100 µg/mL in acetonitrile (ACN), used during the in planta biotransformation experiment, was purchased by Food Risk Management (Oostvoorne, The Netherlands) and stored at 4 °C.

Individual mycotoxin solid standards (1 mg) of ZEN, α-ZEL, β-ZEL, α-ZAL, β-ZAL, and ZAN for LC-MS/MS analysis were purchased from Fermentek (Jerusalem, Israel). All mycotoxin solid standards were dissolved in methanol to a concentration of 1 mg/mL and were storable for a minimum of 1 year at −20 °C. The standard mixture and the ZAN internal standard solution were prepared in methanol, stored at −20 °C, and renewed monthly. LC-MS-grade methanol, formic acid (FA) (99%), and acetic acid (AA) (glacial, 100%) were supplied by BioSolve (Valkenswaard, The Netherlands), while n-hexane (HiPerSolv Chromanorm) and ammonium acetate were obtained from VWR International (Zaventem, Belgium). Ethyl acetate was purchased from Across Organics (Geel, Belgium), and ULC-MS-grade Milli-Q water was used (Merck, Hoeilaart, Belgium).

### 5.3. Biotransformation and Adsorption of ZEN

Both in vitro and in planta biotransformation experiments were set up to investigate the potential of the actinobacteria to degrade and detoxify ZEN. Additionally, an in vitro experiment was conducted with autoclaved cells to evaluate the impact of the adsorption of ZEN.

#### 5.3.1. In Vitro Biotransformation Experiments

The in vitro biotransformation of ZEN was evaluated in two different liquid growth media. LB broth (Merck, Hoeilaart, Belgium) was used as a nutrient-rich growth medium, while minimal medium (MM) with ZEN as the only carbon source was used as a selective medium (Table S2) [63].

The biotransformation experiments were performed in glass tubes filled with 7 mL of growth medium supplemented with 5 mg/L of ZEN. The bacterial suspensions, obtained as previously described, were added to the medium at concentrations of 1% and 2% for LB and MM, respectively, and the aliquots were incubated at 28 °C and 180 rpm. For the biotransformation of ZEN in LB broth, samples of 0.5 mL were taken every 12 h for 3 days, while for the biotransformation in MM, samples were taken every 4 days for a total of 8 days. Control treatments consisted of growth medium with or without 5 mg/L of ZEN, without adding microorganisms. The samples were centrifuged for 5 min at 10,000 rpm, and the pellet and supernatant were stored separately at −20 °C. The experiments in LB and MM were, respectively performed in triplicate and quadruplicate and were repeated twice.

#### 5.3.2. In Vitro Adsorption Experiment

A similar in vitro experiment as above (5.3.1.) was conducted to investigate the adsorption of ZEN to the bacterial cell walls. For this purpose, the bacterial suspensions were autoclaved for 20 min at 121 °C before they were added to the glass tubes filled with 7 mL of LB broth and 5 mg/L of ZEN. The test tubes were incubated at 28 °C and 180 rpm for 24 h, after which samples were taken and the cell-free supernatant was analysed. The experiment was performed once in quadruplicate.

#### 5.3.3. Detached Ear Assay

Ten seeds of spring wheat (*Triticum aestivum* L.) cultivar Tybalt were sown and grown under greenhouse conditions in 3-L pots (15 cm diameter × 30 cm height) until Zadoks GS 65. Subsequently, wheat ears with stalks were cut at a total length of ca. 10 cm. Two spikelets of the detached ear were each treated by point inoculation with 10 µL of the bacterial suspension. Afterwards, both spikelets were each treated with 10 µL of 5 mg/L ZEN in ACN on the same spot as the bacterial suspension. Each treatment consisted of 9 ears and 18 replicates. The control treatments consisted of a PBS solution with or without ZEN standards. The detached ears were separately transferred into falcon tubes and incubated at 25 °C in the dark for 5 days. Afterwards, the treated spikelets were cut off the wheat ear and dried separately at 30 °C for 48 h before storage at −80 °C. Control samples were taken after 0 and after 5 days.

### 5.4. Survival of Actinobacteria on Wheat Ears

A detached ear assay was performed with a similar set-up as above (Section 5.3.3), but with some slight changes. The detached ears were spray-inoculated with 8 mL of the bacterial suspension per six ears. After 25 days of incubation, the top floret of the ear was crushed with sterile sand and PBS + 0.01% Tween 80 (*v*/*v*), and samples were pooled per two. Afterwards, a 10-fold dilution series of the suspension was made in PBS and plated out on TSA. Subsequently, the plates were incubated for 3 days at 28 °C, and eventually the number of colony-forming units (CFUs) of the actinobacterial cells was counted based on their morphology.

### 5.5. Detection of ZEN by UHPLC-MS/MS

The detection of ZEN was performed by a targeted LC-MS/MS method using a Waters Acquity UHPLC system coupled to a Quattro XEVO TQ-S^®^ or XEVO TQ-XS^®^ mass spectrometer (Milford, Manchester, UK).

#### 5.5.1. Sample Preparation and Mycotoxin Extraction

Before analysing the samples from the in vitro trial, they were diluted 50 times to the range of the calibration curve (0–200 µg/L) in a total volume of 1 mL. Every sample was spiked with 30 µL of 2500 µg/mL ZAN internal standard. Five mL of ethyl acetate and 2 mL of hexane were added to 1 mL of the LB samples. After the addition of these solvents, the LB samples were vigorously vortexed and shaken on an overhead shaker for 15 min. Afterwards, they were centrifuged for 10 min at 4000 rpm, and 1 mL of the top ethyl acetate layer was transferred to a glass tube. Subsequently, these samples were evaporated to dryness using a gentle nitrogen stream at 40 °C with the TurboVap^®^ LV (Biotage, Uppsala, Sweden). The samples in MM were directly evaporated to dryness using a gentle nitrogen stream at 80 °C after dilution and internal standard addition.

The dried spikelets from the detached ear assay were first crushed using the Rotor mill pulverisette (Fritsch, Pitsborro, NC, USA). Afterwards, each sample was spiked with 10 µL of 2500 µg/mL ZAN internal standard, and mycotoxins were extracted in 1.5 mL of extraction solvent (ethyl acetate/FA 99/1 (*v*/*v*)). Thereupon, the samples were vigorously vortexed, incubated for 1 h on an overhead shaker, and sonicated. The samples were then centrifuged for 10 min at 4000 rpm, and 1 mL of the extract was transferred to a glass tube. Subsequently, the samples were evaporated to dryness using a gentle nitrogen stream at 40 °C with the TurboVap^®^ LV (Biotage, Uppsala, Sweden).

Prior to LC-MS/MS analysis, all the samples were redissolved in 150 µL of injection solvent (methanol/water 60/40 (*v*/*v*)) and vortexed for 2 min. Eventually, the samples were centrifuged for 10 min at 10,000 rpm in a 0.22 µm Ultrafree-MC centrifugal filter device (Merck, Hoeilaart, Belgium), and 150 µL of the aliquot was transferred to a new injection vial, of which 5 µL was injected into the devices.

After the analysis, the degradation percentage was calculated as follows:

(1) $$\mathrm{Degradation}\;(\%)=\left(1-\frac{c_{s}}{c_{z}}\right)\times 100$$

where *c_s_* represents the average value of the concentration of the unknown or control sample, and *c_z_* represents the average value of the concentration of the ZEN control on day zero.

#### 5.5.2. LC-MS/MS Analysis of In Vitro Degradation

LC-MS/MS analysis of both in vitro experiments was performed in the Centre of Excellence in Mycotoxicology and Public Health. A Waters Acquity UHPLC^®^ HSS T3 (2.1 mm × 100 mm, 1.8 µm) column was used for chromatographic separation (Waters, Manchester, UK). The temperature of the autosampler tray was kept at 10 °C while the column was kept at 40 °C, and two mobile phases were applied for the analysis. Mobile phase A consisted of water/methanol/AA (94/5/1, *v*/*v*/*v*), while mobile phase B consisted of water/methanol/AA (2/97/1, *v*/*v*/*v*) both buffered with 5 mM ammonium acetate. The flow rate was set at 0.4 mL/min with a gradient elution programme that started at 70% mobile phase A with a linear decrease after 4.25 min until 14 min to 27%. After 15.5 min, the concentration of mobile phase A was further decreased to 1%, and initial column conditions were reached again after 17 min using a linear decrease of mobile phase B.

The ESI interface was used in both positive and negative electrospray ionisation mode (ESI+/ESI−) with the following parameters: The capillary voltage was 3.3 kV, the source and desolvation temperatures were 130 °C and 200 °C, respectively, source offset was 60 V, nebuliser pressure was 7.0 bar, collision gas flow was 0.16 mL/min, and nitrogen was applied as the spray gas. LM resolutions 1 and 2 were set at 3.0, while HM resolutions 1 and 2 were set at 15.0. Lastly, ion energies 1 and 2 were 1.0 and 0.4, respectively. The mass spectrometer was operated in multiple reaction monitoring (MRM) mode, and the MS parameters for all the analytes are shown in Table 3.

The limits of detection (LOD) and quantification (LOQ) were determined for each mycotoxin (Table 4) based on the standards set by the International Union of Pure and Applied Chemistry (IUPAC). The LOD was defined as the concentration at which the signal-to-noise (S/N) ratio reached 3, while the LOQ was defined as the concentration at which the S/N ratio reached 10. The accuracy was calculated by dividing the measured value from the calibration plot by the theoretical spiked level.

Data acquisition and processing were performed using MassLynx™ and TargetLynx^®^ version 4.1 software (Waters, Manchester, UK).

#### 5.5.3. LC-MS/MS Analysis of In Planta Degradation

The LC/MS-MS analysis of the in planta trial was performed in the Laboratory of Pharmacology and Toxicology on an Acquity H-Class+ UHPLC coupled to a Xevo TQ-XS^®^ mass spectrometer system (Waters, Manchester, UK) according to Lauwers et al. (2019) [64], with some minor modifications. Chromatographic separation was carried out on an Acquity UHPLC^®^ HSS T3 column (2.1 mm × 100 mm, 1.8 µm) equipped with an Acquity HSS T3 1.8 μm Vanguard pre-column (Waters, Manchester, UK). The temperature of the column oven and autosampler tray were kept at 40 °C and 8 °C, respectively, and two mobile phases were applied for the analysis. Mobile phase A consisted of 1% AA in water and mobile phase B of 1% AA in methanol. The flow rate was set at 0.3 mL/min with the following gradient elution procedure: 0–1 min (95% A/5% B), 1–4.5 min (linear gradient to 40% A/60% B), 4.5–7.8 min (40% A/60% B), and 7.8–8.0 min (linear gradient to 95% A/5% B). The column was then re-equilibrated at 95% A/5% B for 3 min before the next injection.

Mass spectrometer parameters were optimised by infusion of standard working solutions of 100 ng/mL of ZEN and the internal standard ZAN at a flow rate of 10 µL/min in combination with 50% mobile phase A and 50% mobile phase B pumped at a flow rate of 200 µL/min. The following parameters were utilised in ESI mode: capillary voltage: 2.7 kV, source temperature: 130 °C, source offset: 30 V, desolvation gas flow: 800 L/h, source desolvation temperature: 550 °C, nebuliser pressure: 7.0 bar, cone gas flow: 150 L/h, collision gas flow: 0.15 mL/min, LM resolution 1 and 2: 2.8 and HM resolution 1 and 2: 14.0, ion energy 1 and 2: 1.0 and 1.5, respectively. The mass spectrometer was operated in the MRM mode, and the MS parameters for ZEN and ZAN are shown in Table 5.

The limits of detection (LOD) and quantification (LOQ) were determined for ZEN (Table 6) based on the standards set by IUPAC. The LOD was defined as the concentration at which the signal-to-noise (S/N) ratio reached 3, while the LOQ was defined as the concentration at which the S/N ratio reached 10. The accuracy was calculated by dividing the measured value from the calibration plot by the theoretical spiked level.

Data acquisition and processing were performed using MassLynx™ and TargetLynx^®^ version 4.2 software (Waters, Manchester, UK).

### 5.6. Biodetoxification of ZEN: A BLYES Assay to Monitor the Residual Oestrogenicity

Bioluminescent bioreporter *Saccharomyces cerevisiae* BLYES was used to measure the residual toxicity in the samples from the biotransformation experiments. *S. cerevisiae* BLYES is a modified strain transformed with a human oestrogen receptor that produces a bioluminescent signal in response to molecules that bind to the receptor [65]. *S. cerevisiae* BLYES was grown in a modified yeast minimal medium (YMM) lacking leucine and uracil [36] by adding 30 µL of a −80 °C glycerolstock to 25 mL YMM in a sterile Erlenmeyer flask and incubating for two days at 30 °C and 200 rpm to an OD_600_ of 0.6–0.8. In a sterile, flat-bottomed, white 96-well plate (Greiner Bio-One, Vilvoorde, Belgium), 20 µL of each sample was placed in a microwell, after which 200 µL of the grown BLYES culture was added to the wells. Control treatments consisted of blank medium with BLYES culture. The 96-well plate was placed in a Tecan Spark^®^ multimode microplate reader (Männedorf, Switzerland) at 28 °C, and luminescence was measured every hour for 8 h. Bioluminescence intensification (BI) and detoxification percentage were measured as follows:

(2) $$\mathrm{BI}\;(\%)=-1\times \frac{c_{b}-c_{s}}{c_{b}}\times 100$$

(3) $$\mathrm{Detoxification}\;(\%)=\left(1-\frac{{BI}_{s}}{{BI}_{z}}\right)\times 100$$

where *c_b_* represents the average value of relative bioluminescence units (RLU) of parallel negative blank controls after 8 h, *c_s_* the average value of RLU of parallel unknown samples, after 8 h, *BI_s_* the average value of BI of parallel unknown samples and *BI_z_* the average value of BI of parallel ZEN controls.

In addition, *S. cerevisiae BLYR* was used as a control to assess whether samples contained other toxic substances. BLYR is a constitutive strain constantly producing a bioluminescent signal, which will decrease in the presence of toxic substances due to the cytotoxic effect, eventually resulting in cell death. The results from the BLYES assay are only reliable when the bioluminescent signal of the BLYR samples is at least 80% of the signal of the BLYR control (blank medium with BLYR culture); otherwise, cell death has occurred due to the toxic substances in the samples [65].

### 5.7. Statistical Analysis

Statistical analyses were performed in IBM SPSS Statistics version 28. Assumptions regarding normality and homoscedasticity were verified by a Shapiro–Wilk and Levene test, respectively. If assumptions were met, a one-way analysis of variance (ANOVA) followed by a Tukey post hoc test was used to examine significant differences between treatments. When the homogeneity of variances was not met, an ANOVA with Welch correction was used, and a Dunnett’s T3 test was performed for multiple comparisons. When normality assumptions were not met, a Kruskal–Wallis test and a Dunn post hoc test with Bonferroni correction were carried out. All tests were conducted with a significance level of α = 0.05. For plot generation, the R software v.4.3.1 and the package ggplot2 [66] were used.

## Figures and Tables

**Table 1:** Colony-forming units (CFUs) per spikelet and SD for the three actinobacterial strains 25 days post-inoculation (dpi) on wheat ears (*n* = 3 biological replicates).

Treatment	CFU/Spikelet ± SD
LMG19352	1.22 × 10^4^ ± 1.80 × 10^3^ **
R25614	3.43 × 10^3^ ± 3.73 × 10^3 ns^
LMG16995	6.67 × 10^1^ ± 1.15 × 10^2 ns^

ns = not significantly different from 0; ** = *p* < 0.01.

**Table 2:** Concentration ZEN in ng/spikelet and SD for the control treatment analysed with LC-MS/MS on detached wheat ears spiked with 50 ng ZEN/spikelet (*n* = 18 biological replicates, analysed in two separate batches of 9 biological replicates).

Time (Days)	ZEN Concentration (ng/Spikelet) ± SD
0	11.04 ± 2.76
5	1.84 ± 1.35

**Table 3:** MS parameters in multiple reaction monitoring (MRM) mode wereused for the detection of ZEN, ZAN, α-ZEL, β-ZEL, α-ZAL, and β-ZAL in the in vitro samples. *m*/*z* = mass-to-charge ratio.

Analyte	ESI Mode	Quantifier Ion				Qualifier Ion		
		Precursor Ion > Product Ion (*m*/*z*)	Cone Voltage (V)	Collision Energy (eV)		Precursor Ion > Product Ion (*m*/*z*)	Cone Voltage (V)	Collision Energy (eV)
ZEN	+	319.0 > 283.0	40	12		319.0 > 301.0	40	12
ZAN	+	321.2 > 189.1	40	12		321.2 > 303.3	40	8
α-ZEL	+	321.0 > 285.0	40	11		321.0 > 303.0	40	7
β-ZEL	+	321.0 > 285.0	40	11		321.0 > 303.0	40	8
α-ZAL	−	323.2 > 123.0	30	23		323.2 > 305.2	30	23
β-ZAL	−	323.2 > 189.1	30	23		323.2 > 305.2	30	23

**Table 4:** Limits of detection (LOD), limits of quantification (LOQ), and accuracy for analysis of each mycotoxin in LB broth and MM.

Analyte	LOD (µg/L)	LOQ (µg/L)	Accuracy (%)
ZEN	5	9	99.60
α-ZEL	10	20	106.33
β-ZEL	14	29	102.37
α-ZAL	27	55	96.49
β-ZAL	13	26	109.39

**Table 5:** MS parameters in MRM mode that were used for the detection of ZEN and ZAN in the in planta samples. *m*/*z* = mass-to-charge ratio.

Analyte	ESI Mode	Quantifier Ion				Qualifier Ion		
		Precursor Ion > Product Ion (*m*/*z*)	Cone Voltage (V)	Collision Energy (eV)		Precursor Ion > Product Ion (*m*/*z*)	Cone Voltage (V)	Collision Energy (eV)
ZEN	-	317.1 > 175.0	15	25		317.1 > 130.8	15	30
ZAN	-	319.1 > 275.0	20	22		319.1 > 205.0	20	20

**Table 6:** Limit of detection (LOD), limit of quantification (LOQ), and accuracy for analysis of ZEN in wheat ears.

Analyte	LOD (ng)	LOQ (ng)	Accuracy (%)
ZEN	0.067	0.222	100.74

## Data Availability

The original contributions presented in the study are included in the article/Supplementary Materials, further inquiries can be directed to the corresponding authors.

## Supplementary Materials

The following supporting information can be downloaded at: https://www.mdpi.com/article/10.3390/toxins16060253/s1, Table S1: Detoxification (%) of 5 mg/L ZEN in LB after 24 h incubation at 28 °C and 180 rpm due to adsorption to autoclaved cells; Figure S1: Percentage degradation (A) and detoxification (B) of 5 mg/L ZEN by three actinobacterial strains in LB broth between 0 and 72 h after inoculation analysed with LC-MS/MS and BLYES assay, respectively. The values represent means ± SD. of three replicates of the repeated experiment; Figure S2: Percentage degradation (A) and detoxification (B) of 5 mg/L ZEN by three actinobacterial strains in MM with ZEN as the only carbon source at different time points analysed with LC-MS/MS and BLYES assay, respectively. Samples were incubated at 28 °C and 180 rpm for 12 days. The values are the means ± SD of four replicates of the repeated experiment; Table S2: Composition of minimal medium (MM) per litre.
