# Supplementary material for: Actinobacteria as Promising Biocontrol Agents for In Vitro and In Planta Degradation and Detoxification of Zearalenone
Source: Toxins (Basel). 2024 May 28;16(6):253. doi: 10.3390/toxins16060253 (PMC11209476; doi:10.3390/toxins16060253)
Supplement: Supplementary file 1 [file toxins-16-00253-s001.zip › toxins-2997141-supplementary.pdf]

Supplementary Materials: Actinobacteria as Promising Biocontrol Agents for In Vitro and In Planta Degradation and Detoxification of Zearalenone

Larissa De Troyer, Noémie De Zutter, Sarah De Saeger, Frédéric Dumoulin, Siska Croubels, Siegrid De Baere, Leen De Gelder and Kris Audenaert

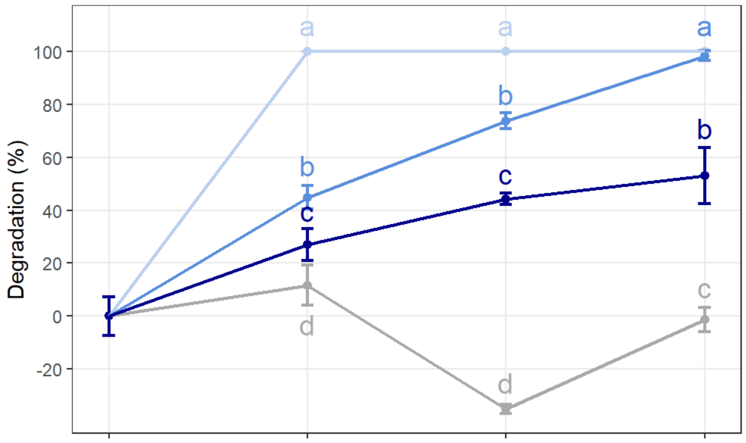

(**A**)

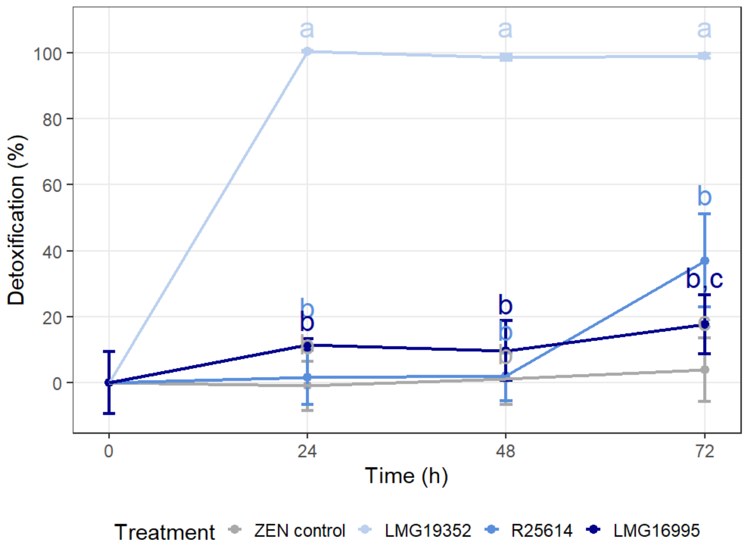

(**B**)

Figure S1: Percentage degradation (**A**) and detoxification (**B**) of 5 mg/L ZEN by three actinobacterial strains in LB broth between 0 and 72 h after inoculation, analysed with LC-MS/MS and BLYES assay, respectively. Samples were incubated at 28 °C and 180 rpm. The values represent means ± SD of three replicates of the repeated experiment. Values not sharing common letters at a certain time point are significantly different ($\alpha = 0.05$). When no significant differences were observed between treatments at a certain time point, significance letters were omitted.

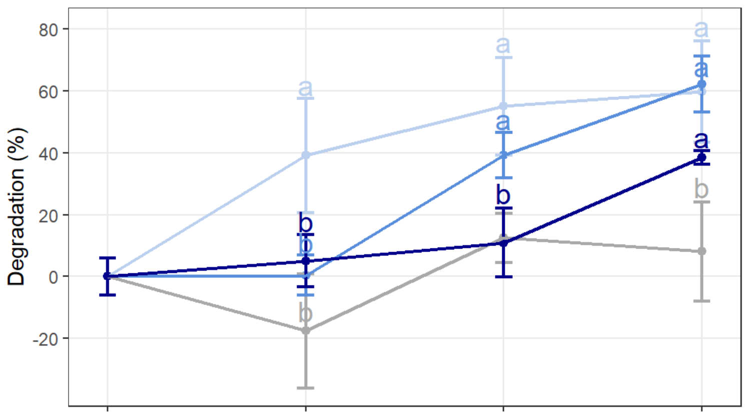

(**A**)

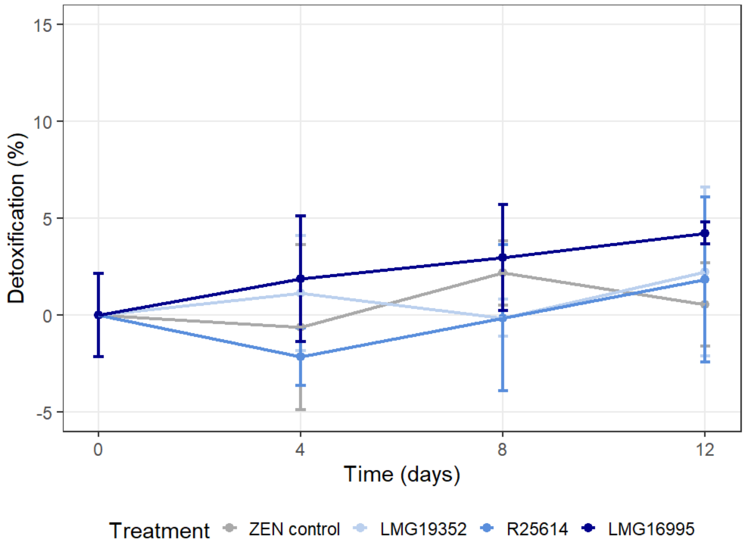

(**B**)

Figure S2: Percentage degradation (**A**) and detoxification (**B**) of 5 mg/L ZEN by three actinobacterial strains in MM with ZEN as the only carbon source at different time points, analysed with LC-MS/MS and BLYES assay, respectively. Samples were incubated at 28 °C and 180 rpm for 12 days. The values are means ± SD of 4 replicates of the repeated experiment. Values not sharing common letters at a certain time point are significantly different (α = 0.05). When no significant differences were observed between treatments at a certain time point, significance letters were omitted.

Table S1: Detoxification (%) of 5 mg/L ZEN in LB after 24 h incubation at 28°C and 180 rpm due to adsorption to autoclaved cells.

Treatment	Detoxification (%)
ZEN control	2.8 ± 1.1
LMG19352	0.4 ± 1.0
R25614	0.6 ± 2.5
LMG16995	0.4 ± 1.6

Table S2: Composition of minimal medium (MM) per liter.

Solution	Reagentia	Concentration stock solution (g/L)	Volume (mL)
Stock A	Na_2HPO_4	141.960	10
	KH_2PO_4	136.090	
Stock B	$(NH_4)_2SO_4$	100.000	3
Stock C	$MgSO_4$	9.600	5
Stock D	$CaCl_2.H_2O$	1.150	5
Stock E*	Na_2EDTA	0.640	5
	$FeSO_4.7H_2O$	0.550	
	$ZnSO_4.7H_2O$	0.230	
	$MnSO_4.H_2O$	0.340	
	$CuSO_4.5H_2O$	0.075	
	$CoCl_2.6H_2O$	0.038	
	$(NH_4)_6Mo_6O_{24}.4H_2O$	0.025	

* Stock E was filtersterilised instead of autoclaved
